# Continuity and change in local immigrant policies in times of austerity

**DOI:** 10.1186/s40878-017-0067-x

**Published:** 2018-02-06

**Authors:** Maria Schiller, Sarah Hackett

**Affiliations:** 1 Max Planck Institute for the Study of Religious and Ethnic Diversity, Hermann-Föge-Weg 11, D-37073 Göttingen, Germany; 2 Reader in Modern European History, Bath Spa University, Newton St. Loe, Bath, BA2 9BN UK

**Keywords:** Immigrant policy, Crisis, Cities, Europe

## Abstract

European cities are increasingly being recognised for the role they play in devising and implementing their own migration, integration and diversity policies. Yet very little is known about the local dimension of immigrant policymaking in crisis contexts. This introductory piece offers a rationale for analysing city-level immigrant policies in times of crisis and the salience of using crisis as a metaphor for the state of things, and outlines key scholarly works, debates, concepts and theories. It provides a range of historical and contemporary examples and considerations, and introduces an empirical city case study that is published as part of this mini-symposium. It argues that a crisis lens leads to a systematic understanding of local-level immigrant policymaking in recent and contemporary Western Europe. The mini-symposium’s focus and findings should be relevant to both on-going academic and policy debates.

## Introduction

After several decades without profound political or economic crises, Europe is currently confronted with several developments that challenge assumptions of constant stability and linear growth. These include the 2008 financial crisis, the annexation of Crimea and Eastern parts of Ukraine by Russian forces, the entry of extreme nationalist forces into several EU countries’ governments, the on-going war in Syria and the displacement of large parts of its population, as well as incidents of international terrorism in European metropolises. Some of these developments profoundly impact migration patterns into, and configurations of diversity in, Europe, and it is their ramifications on the policy area of immigrant incorporation that this mini-symposium focuses on. The large numbers of asylum seekers arriving to Europe in 2015 for instance put both solidarity among EU members and the Schengen Agreement to the test, as a number of states have declared their unwillingness to accommodate asylum seekers and have closed their borders to them.

In view of the current refugee flows from Afghanistan, Iraq, Syria and a range of African countries, migration has once again become an intrinsic marker of crisis and instability in media representations, political statements, and in popular and social settings and contexts. In the area of immigrant incorporation, the purported crisis of multiculturalism of the late 1990s and 2000s provides an example of representing immigrant policy as being “in crisis”, this time focusing on questions of diversity and on adequate responses to difference in increasingly diversified societies. Indeed, the crisis metaphor is highly overused in assessments of migration and immigrant incorporation, and migration is often equated with the crisis itself. While we focus on crisis discourse and its repercussions on local integration policies, this discourse in some cases may build on a widely shared experience of crisis, whilst in other cases it is a narrative that is strategically used to inform people’s perceptions of reality.

Studying the effects of crisis and representations of crisis on the policy area of immigrant incorporation is therefore urgently needed in contemporary migration scholarship, and this mini-symposium represents a step forward in this area. Crisis and representations of crisis can provide a rupture for ways of working, stretch existing resources, alter the priorities of policymakers, and trigger the transformation of existing practices. Over the past decades, the policy area of immigrant incorporation has experienced a number of conceptual changes, and some adaptations of resources and practices, often based on a discourse of crisis. Yet, overall, this policy area has become increasingly established and many European countries today, for example, have a dedicated ministry working on immigrant policy. Also, the greater value ascribed to this policy area is reflected in the various exchanges organised across cities and countries on immigrant incorporation. All this points to the consolidation of a policy field and it is unclear how recurrent crisis and representations of crisis are correlated to such patterns of institutionalisation and path-dependency. Recent cuts to human resources and the dismantling of institutional structures dedicated to immigrant and diversity policies provide a puzzle that calls for investigations into the ways in which different representations of crises play a role in shaping immigrant incorporation efforts.

Crisis rhetoric in relation to migration is common in national and EU-level debates and deliberations, but it is cities that are dealing with the bulk of issues surrounding immigrant incorporation policy. In the context of the current inflow of asylum seekers, it is them who are becoming home to a large share of these immigrants, are perceived to be centres of economic and social opportunity, and are implementing a plethora of programmes and initiatives in an attempt to offer resettlement, accommodation, healthcare, language courses, and an overall chance of incorporation.

The aim of such research on representations of crisis and responses to migration is not to discuss and thereby reproduce and reinforce “migration as crisis” discourses, but to analyse the ramifications that representations of crisis can have on local conceptions, structures and agencies surrounding immigrant incorporation. This mini-symposium intends to provide stimulus for further research into city-level immigrant policies and crisis. There may be a continuity of immigrant policy despite representations of crisis, as for instance this mini-symposium’s empirical case study, located in Italy, shows (Caponio & Donatiello, 2017). Future research could reveal whether this is a general pattern, or whether other case studies find that an economic downturn and austerity measures can have a destructive and detrimental effect.^1^ Furthermore, whilst we take representations of the economic crisis as one example of a crisis discourse, it is important to acknowledge that other representations of crisis, be they political, or regarding asylum or the on-going migrant crisis specifically, are certain to also have ramifications for the development and implementation of city-level immigrant policies in Europe.

### State of the art

Although as Lakoff and Johnson remind us, metaphors have an important function of allowing us to use what we know about our physical and social experience to understand and classify new experiences, (Lakoff & Johnson, 2003) they can also have the detrimental effect of creating false truths. We acknowledge the important work that has been done by scholars to reveal populist uses of crisis discourse in assessments of migration and immigrant incorporation. In their seminal work, research has shown how common discourses on race and migration often amount to nothing more than myths and are actually reinforcing inequality and prejudice (Finney, 2009; Lindley, 2014). This literature, however, has not thus far sufficiently discussed how the use of the crisis metaphor affects policies and practices in Europe.

The manner in which European cities have become important players in devising and implementing immigrant policies has been progressively recognised, both regarding recent refugee flows and more settled migrant communities. Several explanations for the changed role and self-confidence of cities have been offered, such as the promotion of local responses to migration and diversification by European Union institutions, the evolution of city networks, the questioning of national-level policy frameworks, and the immediate pressures cities face to respond to local diversity. Academic scholarship has now gone some way towards analysing local-level immigrant policymaking, and how it can and does deviate from national policy directives and legislation; the importance of cities in putting integration policies into effect; and the extent to which a city’s relationship with a diversified local population can be influenced by its policies, economy and history (Alexander, 2003; Glick Schiller & Çağlar, 2011; Hepburn & Zapata-Barrero, 2014; Nicholls & Uitermark, 2013; Penninx, Kraal, Martiniello, & Vertovec, 2004).

Representations of crisis in the context of the recent economic slump, as well as past representations of economic crisis, provide a pertinent example for a systematic discussion regarding the devising and implementation of city-level immigrant policies in light of a crisis discourse. Despite impacting different parts of Europe in unequal ways, representations of economic crisis are having deep ramifications at political and policy levels. This has been recognised within national contexts, (Bevelander & Petersson, 2014; Pastore, 2014) and a recent study carried out by Patrick Ireland has provided convincing evidence that an economic crisis discourse can have ramifications on local integration policies as well (Ireland, 2017). Furthermore, it has long been argued that an economic recession leads to a more restrictive political approach to migration (Ghosh, 2013; Hollifield, 1992). Indeed, regarding the Great Depression of the 1930s, the most severe economic crisis to have ever affected the industrialised Western world, attention is often called to the fact that both the United States and Europe implemented a range of control measures in an attempt to protect the domestic labour market (Camayd-Freixas, 2013; Hammar, 1985). Similarly, it has frequently been pointed out how during the Asian financial crisis of 1997–1998 several countries in the region demonstrated a preference for domestic workers and enforced expulsion policies (Koser, 2009). Yet the reality has been far more complex.

The 1973 oil crisis and subsequent economic downturn, for example, did result in Northern European countries halting the recruitment of foreign workers, and this period is in fact perceived as being the turning point during which many European countries implemented their first restrictive immigration policies (Gubert, 2014; Lucassen, 2005). Yet simultaneously, these years marked a shift towards immigrant policies aimed at promoting integration and multiculturalism (Castles & Miller, 2003; Vermeulen & Penninx, 2000; Wihtol de Wenden, 2011). Similarly, the 2008 financial crisis and its aftermath have led to a wide range of migration policy responses across the EU, with states implementing restrictive or liberal policies, or a combination thereof. Policies have included Spain’s restrictions to family reunification and voluntary return schemes, Greece’s voluntary assisted return programme for illegal immigrants, Britain’s combination of curbing labour immigration whilst favouring high-skilled migrants, Portugal’s training opportunities in the area of immigrant entrepreneurship, and Sweden’s liberalisation of its labour migration system (Devitt, 2014; Koehler, Laczko, Aghazarm, & Schad, 2010; Triandafyllidou, 2013).

The city-level response to migrant populations in periods of economic recession has not been investigated or documented to the same extent though some information is available. Regarding Britain, for example, it has been argued that the economic downturn of the late 1950s caused race riots in both London’s Notting Hill district and Nottingham. The recession of the early 1960s led to anti-immigrant sentiments in the West Midlands constituency of Smethwick, which, in turn, resulted in what has often been perceived as Britain’s most racist election (Meyers, 2004). In the Netherlands, Rotterdam pursued an integration policy in an attempt to help its migrant communities who had been hit hard by the recession of the 1970s. In Germany, the cities of Bremen and Nuremberg remained fully committed to migrant integration during the 1980s, despite their severe encounters with economic hardship (Hackett, 2013; Ireland, 2004; Scholten, 2013). Likewise, it is clear that European cities’ approaches to migration, integration and diversity have been affected by the 2008 financial crisis and the subsequent period of austerity, albeit to differing degrees. In some contexts, the economic crisis was an intermediary moment or an ‘episode’. In other cases, the economic crisis and austerity measures may have started a long-term process of transformation due to the crisis denoting a context or a more ‘endemic’ condition in which people live (Vigh, 2008).

Cities have been marked by austerity measures and spending cuts, especially in countries that implemented substantive constraints in public spending, as well as by a widespread anti-immigrant backlash. Within the context of the global economic crisis, migration and integration policies have often been perceived as being both unpopular and superfluous (EUROCITIES, 2015; Ponzo et al., 2013). In the context of the 2009 economic crisis, several local equality and diversity offices in the UK lost some of their human resources. For instance, in Leeds, the equality and diversity unit lost several of its staff due to cuts in personnel resulting from the implementation of subsequent austerity measures (Schiller, 2016). Yet, despite these constraints, many European cities have persevered and have awarded a significant level of importance to pursuing the integration of their migrant communities (Collett, 2011). Over the last few years, for example, Barcelona’s immigration department has enjoyed an increased budget, Toulouse has re-energised and expanded its integration debate, and Oslo’s business community and universities have joined forces in an attempt to foster a welcoming culture in the city. Ghent has committed itself to combatting discrimination in the areas of employment, housing and education, Oulu and Tampere have implemented new measures concerning equality and integration, and Riga has cited integration as being an official priority, and is working towards becoming a multicultural and tolerant city (EUROCITIES, 2015). Whilst such a brief snapshot into various European cities’ political approaches to migration, integration and diversity during times of economic crisis is available, we still lack an in-depth understanding of how exactly this relationship influences and shapes the policymaking process. Indeed local structures for governing migration, integration and diversity are currently being reorganised, reduced or dissolved, and local policies are being adapted.

### The Italian case study and the mini-symposium’s contribution

The mini-symposium showcases an empirical case study from the city of Turin, which is characterised by a distinct immigration history, minority population, approach to immigrant settlement and inclusion, and political, economic and social profile. Overall, city-level immigrant policies have persisted more, and experienced limited changes and revisions, in Turin. Caponio and Donatiello’s article analyses the emergence of interculturalism within the context of the current economic crisis at the neighbourhood level in the city of Turin. They argue that, contrary to what might be expected, and despite financial cuts and scarce resources, immigrant integration policy paradigms have witnessed a substantial degree of continuation as a result of local perseverance and determination, social contexts, structural features and organisational patterns.

The recent economic crisis and on-going austerity measures thus call into question the implicit assumption in much scholarly work on local immigrant policymaking that this is a steadily growing policy area and that the presence of diversity in the city is increasingly accepted. It challenges us to conceive of immigration politics through a more dynamic perspective and to think through established binaries of continuity and rupture, and growth and decline. How and when do cities reduce their personnel or change their policy offer? How and under which circumstances do new policies arise? How and when are the perceptions of different groups of migrants or their self-images altered? How can the change of the material configuration of immigrant policies be explained? We focus here on economic crisis as one possible form of crisis narrative, all the while acknowledging that other forms of crisis and crisis narratives may have similar or different, weaker or even more profound, effects on local immigrant policies.

Furthermore, beyond this case study, the mini-symposium contributes to a research framework that sheds light on how and to what extent cities’ migration, integration and diversity policies change as a consequence of crisis and crisis discourse. Thus, it hopes to provide the foundation for a more extensive consideration of the relevance of crisis and crisis discourses for immigrant incorporation. More generally, it contributes to a systematic understanding of local-level immigrant policymaking in recent and contemporary Western Europe. In doing so, it acknowledges the recent shift in focus from the “traditional national models of integration” to the local aspect of migrant integration. It recognises the local dimension of migration, integration and diversity policies, and shares the observation that cities, and indeed neighbourhoods, have become increasingly active in devising and implementing immigrant policies. Furthermore, it demonstrates that city-level policies not only often reflect local concerns, challenges and contexts, and thus constitute a political approach to migration in their own right, but that they are also inherently influenced by national and global economic developments. It thus confirms the importance of a multi-level governance perspective, which acknowledges and appreciates the interrelationship between the global, national, regional, and local realms of policymaking, as has been recently highlighted (Caponio & Jones-Correa, 2017; Scholten & Penninx, 2016).

Finally and importantly, this mini-symposium offers a dynamic perspective on city-level immigrant policies that recognises the vulnerability of local policies and the possible dismantling of existing city policies, structures and practices due to crisis as well as discoursive uses of the crisis metaphor. Debates on the role of the local level in the area of immigrant incorporation have gradually gained prominence in the migration studies literature. An analysis of the adaptation of local immigrant policies both complements and advances these discussions through a previously unexplored crisis perspective.

## Footnotes

1 During the 1990s, the city of Newcastle upon Tyne in the UK provisioned for ethnic minority businesses in the hope they would help regenerate an area of the city in decline. Similarly, the German city of Bremen has perceived ethnic minority businesses as having the potential to revive the city’s post-industrial economy by developing foreign trade and new markets, and building upon its role as an international port and foreign trade centre. See Hackett (2013). *Foreigners, minorities and integration: the Muslim immigrant experience in Britain and Germany*. Manchester: Manchester University Press.
